# Supplementary material for: Splice-Junction-Based Mapping of Alternative Isoforms in the Human Proteome
Source: Cell Rep. Author manuscript; Available in PMC 2020 Jan 15. (PMC6961840; doi:10.1016/j.celrep.2019.11.026)
Supplement: 2 [file NIHMS1546469-supplement-2.zip › DF1/PXD010154/Lung/Lung_36_PIH1D1_MQVGGGAAVEAWAGVSTPTLPATACLFPLCHP.pdf]

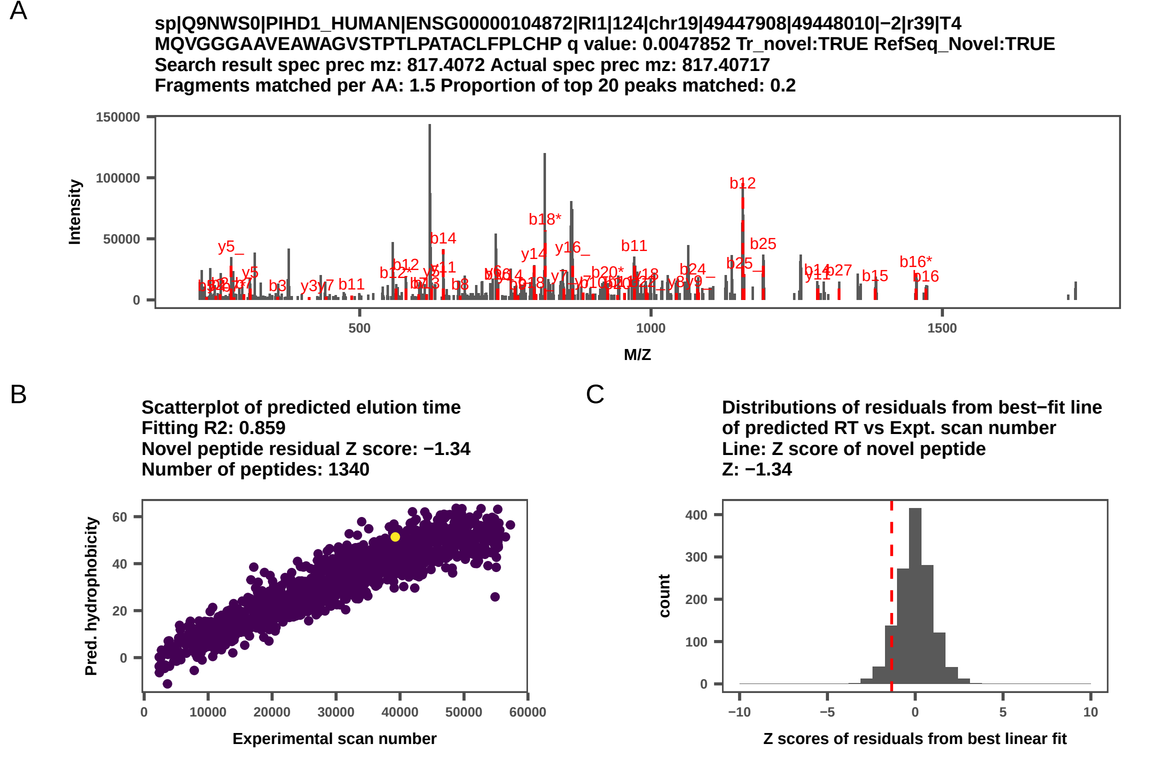
A
sp|Q9NWS0|PIHD1_HUMAN|ENSG00000104872|RI1|124|chr19|49447908|49448010|−2|r39|T4
MQVGGGAAVEAWAGVSTPTLPATACLFPLCHP q value: 0.0047852 Tr_novel:TRUE RefSeq_Novel:TRUE
Search result spec prec mz: 817.4072 Actual spec prec mz: 817.40717
Fragments matched per AA: 1.5 Proportion of top 20 peaks matched: 0.2
Intensity
150000
100000
50000
0
M/Z
500
1000
1500
b12
b18*
b14
y5_
y14
y16_
b11
b25
b25_
b12*
b16*
b16
b15
b27
B
Scatterplot of predicted elution time
Fitting R2: 0.859
Novel peptide residual Z score: −1.34
Number of peptides: 1340
Pred. hydrophobicity
60
40
20
0
0
10000
20000
30000
40000
50000
60000
Experimental scan number
C
Distributions of residuals from best−fit line
of predicted RT vs Expt. scan number
Line: Z score of novel peptide
Z: −1.34
count
400
300
200
100
0
−10
−5
0
5
10
Z scores of residuals from best linear fit
